# The relationship between resilience, anxiety, and depression in Chinese collegiate athletes

**DOI:** 10.3389/fpsyg.2022.921419

**Published:** 2022-08-12

**Authors:** Chengjie Lyu, Rong Ma, Ronald Hager, Dave Porter

**Affiliations:** ^1^School of Physical Education, Guangzhou Sport University, Guangzhou, China; ^2^Athletic Department, Brigham Young University, Provo, UT, United States; ^3^Exercise Science, College of Life Sciences, Brigham Young University, Provo, UT, United States

**Keywords:** anxiety, depression, resilience, collegiate athletes, positive psychology

## Abstract

Anxiety disorder is becoming increasingly prevalent across college campuses, particularly among collegiate athletes who face different stressors and challenges than their non-athlete peers. Anxiety and depression are well documented comorbidities. Furthermore, the number of Chinese collegiate athletes suffering from anxiety and depression is increasing. Psychologists have recently begun to take a more prophylactic approach to defend against these disorders through resilience education. Resilience education focuses on developing greater capacity to cope when mental and emotional challenges arise. The purpose of this study was to analyze the relationship between resilience, depression, and anxiety in Chinese collegiate athletes. This study provides additional evidence of the value of incorporating an effective resilience educational module amongst collegiate athletes.

**Research design:**

The study employed a survey research design to explore the complex relationship between depression, anxiety, and resilience for collegiate student athletes.

**Data analysis strategy:**

Structural Equation Modeling (SEM) was employed to account for any measurement error between the observed items (responses on the instruments) and the latent variables (anxiety, depression, and resilience). The theoretical hypothesized relationship for this study is an inverse relationship between anxiety and depression, on the one hand, and resilience on the other hand. The SEM statistical analysis from this study supported this theoretical model.

**Effective sample size:**

599 collegiate athletes from six different universities in the province of Guangdong, China P.R. participated in this study.

## Introduction

Anxiety and depression are becoming increasingly prevalent in many societies. It is well established that anxiety and depression are intricately connected and can simultaneously affect adolescent and young adult populations, specifically, college students (Bitsko et al., 2018). Substance and alcohol abuse, along with challenges in academic, social, and family life may exacerbate these conditions (Tubman and Windle, 1995; Birmaher and Brent, 2007). A recent survey indicated that over 300 million people around the world currently suffer from depression, and this number increased by 18% between 2005 and 2015 (World Health Organization, 2017). Globally, 18% of the population every year suffer from anxiety disorder (Anxiety and Depression Association of America, 2022). That number is slightly higher amongst Chinese college students, approximately one-fifth of whom report experiencing psychological difficulties such as anxiety, stress, and depression (Wei and Sang, 2017; Liu et al., 2019).

The Diagnosis Statistical Manuel of Mental Disorders 5th edition (DSM-5) and American Psychiatric Association (APA) define anxiety as an excessive worry and fear about a plethora of life events and negative memories that also are accompanied with physical tension and vigilance (Ruscio and Ruscio, 2004; Craske and Waters, 2005; Moffitt et al., 2007; American Psychiatric Association, 2013). As Fullerton (2018) found, college athletes often experience anxiety resulting from an imaginal stress or a negative anticipation. For example, college athletes may imagine themselves losing an important match, or visualize themselves suffering an injury during the competition. The common stressors may make collegiate athletes even more prone to experiencing anxiety and depression. In addition, Cowden et al. (2014) argue that the various stressors coming from the high expectation placed on athletic performance may cause athletes to perceive competitive situations as threatening, and to respond to these situations with increased feelings of apprehension and tension.

Psychologist Rawson et al. (1994) have similarly shown that people who suffer from depression also experience increased levels of anxiety. The APA and previous substantial research indicate that anxiety and depression are remarkably linked together (Castonguay and Oltmanns, 2013; Naragon-Gainey et al., 2016; Weber et al., 2018).

Depression may manifest as a perception that losing important resources in life are too overwhelming that exceeds the individuals’ ability or competencies to adapt such emotional trauma (Beck and Bredemeier, 2016). For instance, people need emotional and substantial support from their families, close friends, romantic partners, or identity groups to satisfy their spiritual, mental, and biological necessities (Beck and Bredemeier, 2016). People with depression tend to focus on the belief that if they lose these support resources they will not be able to satisfy their needs (Beck and Bredemeier, 2016). Neuroscientists and psychobiologists indicate some people have a hard time mitigating the symptoms of depression because these affected individuals are being too negative on themselves (Pankspp, 2011; Pankspp et al., 2011). Pankspp, also states that people’s emotional system consists of “seven networks: seeking, rage, fear, sexual lust, maternal care, separation-distress panic/grief, and joyful play” (Pankspp, 2011; Pankspp et al., 2011). Individuals who suffer from depression often tend to exhibit high levels of separation-distress panic/grief in the emotional system. For example, if an athlete keeps ruminating on a disappointed experience for a long time after a tough loss in a competition, the athlete may start developing symptoms of depression.

Secades and his colleagues claim that frequent occurrences of stressful situations in competition cause many athletes to experience higher levels of anxiety, disappointment, depression, and withdrawal from sports (Secades et al., 2016). Rao and Hong (2015) also indicate depression and suicidal thoughts are tangible concerns for current college student athletes. Rao and Hong further state that a better understanding of risk factors for depression is the prerequisite to effectively manage psychological despondency (Rao and Hong, 2015). Hence, mental health staff, sport psychologists, and coaches must help collegiate athletes to understand the prevalence and risk factors associated with depression among athletes before any integrated psychological intervention takes place.

In the past two decades, the People’s Republic of China (PRC) has started focusing more on aspects of mental health education, and research and intervention options for Chinese college students (Liu et al., 2017). In 2005, the PRC government emphasized the importance of developing sound psychological health education and consultation services. This effort has resulted in recruiting part-time or full-time instructors specifically for college students’ mental health education (Liu et al., 2017).

From the beginning of the 21st century, China has been gradually integrating sport and education at the college or university level, resulting in increasing numbers of collegiate athletes in China (Hua, 2006; Huang, 2008; Zhang, 2014). A lot of these student athletes choose to go to the universities to become collegiate athletes (Graceffo, 2022). Indeed, every year about 100,000 student athletes from mainland China graduate from various sport centers across the country, but only 3% or roughly 2700 of this population are admitted to the professional/national teams. However, according to the authors’ best knowledge, very few studies have been conducted to examine Chinese collegiate athletes’ mental health and well-being in this current climate.

Previous research findings indicate that the number of collegiate athletes suffering from depression and anxiety is growing, due in part to student athletes who become injured and are required to split their attention between their academic and athletic or rehabilitative endeavors (Yang et al., 2007). Chinese collegiate athletes may experience even more challenges related to academic performance because their backgrounds—beginning from childhood— generally tend to focus on sports training, as opposed to academics (Hong, 2004). Due to historical and political influences, athletic performance (especially for elite or international competitive sports) is treated as an opportunity to demonstrate the capability of the Chinese people, and to display the superiority of Chinese government programs and philosophies (Hong et al., 2005; Cha, 2009). Individuals are selected early (from ages 6–13 years) by Provincial and National coaching groups to become athletes and live and train full time for their sports at National or Provincial sport centers (Hao, 2004; Hong, 2004; Hong et al., 2008).

The framework and training methods employed at these Provincial and National sport centers are heavily influenced by Chinese cultural values, one of which is “that success in almost any endeavor calls for total concentration and the focusing of all energies on the task at hand. It is taken for granted that anyone who has achieved greatness in any activity has done so because of their complete devotion” (Hong, 2004, p. 348). Most elite Chinese athletes begin training at a young age, with long hours of training, often six days a week, with the hope of one day competing for Provincial Teams, and ultimately, the opportunity to represent the PRC in the Olympic Games (Hong, 1998; Hong et al., 2005). The national sport centers throughout the country provide elementary and high school equivalent academic programs. However, research has shown that the attention to education, time for study, and quality of learning are insufficient to prepare young athletes for future career development or to be successful in a regular university setting (Gao, 2015). Research further indicates that many elite athletes in China are overworked and deprived of education and healthcare opportunities. These athletes have little chance to succeed outside of their sport (Hong, 2004).

Depending on their athletic preparation and background, many Chinese athletes are eventually admitted to universities by way of special government policies. This ensures government support for the athletes who have historically excelled in sports and allows them to receive a higher education (Gao, 2015). Additionally, through intercollegiate competition in China, most Chinese universities provide significant scholarships, stipends, and lowered university entrance exam standards to recruit elite athletes. Once at the university, athletes train to compete in the Chinese National Varsity Games (Hu and Wang, 2008).

Many elite Chinese athletes are eventually admitted to college, but their poor educational background and inadequate vocational development from a young age create difficulty in adjusting to the academic rigors of a university. Athletes tend to have a poorer academic record compared with their non-athlete counterparts, and lower self-esteem because of underachievement in academia. As a result, many athletes believe they are not good at anything other than their sport (Hong, 2004).

Inherent in any competitive sport is the fact that most collegiate athletes will suffer defeats throughout the year, as the reality is that only one athlete, or one team, can ultimately win in any given season, while all others must lose, at some point, to end their seasons (Wolanin et al., 2015). It is easy, and common, for most athletes to attribute their losses to their declined athletic performance or even a devastating (choking) athletic performance (Wolanin et al., 2015). From a psychological standpoint, athletes may experience symptoms of depression and have negative self-perceptions, and feelings of despondency or uselessness if they feel that they performed inadequately in the contests, or if the outcome of the competition did not match their expectations. Hammond et al. (2013) conducted a study on the prevalence of depression symptoms in collegiate swimmers in Canada who either lost, or perceived that they failed in terms of their performance, and how this may have influenced these athletes’ emotional status. It was discovered that 68% of athletes from the study had major depressive symptom history and female athletes notably felt more depressed than male athletes. The authors also found that after competition, 34% of athletes had elevated depression scores on the Beck Depression Inventory (BDI-II) and elite athletes from the study (those whose times qualify for World Championships or Olympics) reported 2 times higher depression scores than other swimmers who could not qualify for World Championships or the Olympics (Hammond et al., 2013). The study concluded that athletes, especially high-performance athletes, are likely to experience depression symptoms, especially if the performance results are below their expectations (Hammond et al., 2013). There is obviously inherent stress associated with athletic performance, and for many collegiate athletes there are myriad life-events to balance with the demands of being an athlete. Post-graduation employment opportunities, family issues, romantic relationships, injuries, and other stressors in life may often have compounding effects on depression risk. Like any other athletes, Chinese collegiate athletes are also vulnerable to anxiety, depression, and other psychological distress (Hong, 2004; Lu et al., 2016; Rice et al., 2016; Sun et al., 2020).

Researchers in the fields of neuroscience and psychology have found that those who have a strong sense of resilience; emotionally and mentally, can cope with depression and anxiety much more effectively (Charney and Nemeroff, 2004; Kent, 2014; Everly et al., 2015; Tunariu et al., 2017; Coulombe et al., 2020). The basic definition of resilience comes from mammalian adaptation literature, and refers to the life-preserving ability of an organism to adjust, and even thrive when confronted by repeated environmental vicissitudes (Kent, 2014). For humans, resilience is the mental capability that helps individuals withstand physical or emotional difficulties (Charney and Nemeroff, 2004; Kent, 2014; Everly et al., 2015).

Resiliency literature has been studied in many scientific fields. For instance, in the field of psychiatry, humans use resilience training to bolster biological and psychological strengths to manage change successfully (Flach, 1988). In developmental psychopathology, resilience refers to the ability to deal with threats and difficulties while keeping an integrated sense of inner self (Garmezy and Masten, 1986). In the field of human development, resiliency was conceptualized as the ability to withstand or even thrive when faced with adversity (Werner and Smith, 2001).

Resilience is the ability to experience problems, defeat, embarrassment, challenges, and not only return back to the level where we were before, but to achieve greater success, happiness and mental strength (Grotberg, 2003; Fletcher and Sarkar, 2013; Everly et al., 2015). An old Chinese idiom “Failure is the mother of success” (Bossman, 2015, p.130), suggests that failure is necessary to produce success. Relatedly, the concept of resilience recognizes that defeat, tragedy, and stress create opportunities to learn and adapt in order to overcome, recover, and grow (Charney and Nemeroff, 2004; Shi et al., 2019).

Resilience can help individuals alleviate or eliminate debilitating symptoms of anxiety and depression. Through resilience the triggers for anxiety and depression can ultimately become opportunities to overcome symptoms and improve performance. Over the last several decades, mental health research has shifted the focal point away from a pathological-orientation in order to emphasize the role of resilience in health (Kumpfer, 1999; Rutter, 2000; Connor and Davidson, 2003).

Based on the potential of resilience to help individuals cope with depression and anxiety, the aim of this study is to investigate the relationship between resilience, anxiety, and depression among Chinese collegiate athletes. It is hoped that the findings from this study will contribute to efforts to develop effective resilience education curriculum, such as emphasizing positive psychology learning for collegiate sports programs that can be used to help athletes overcome anxiety and depression.

## Materials and methods

### Participants

The authors from this study reached out to approximately 1300 collegiate athletes from six different universities: Guangzhou Sport University, Lingnan University, Shenzhen University, South China Normal University, South China University of Technology, and Sun Yat-sen University, in the province of Guangdong, China P. R. A total of 599 participants (effective sample size: 599) from these universities completed all the questionnaires. This study included participants/athletes from the following sports: tennis, soccer, volleyball, sailing, wrestling, fencing, track and field, gymnastics, martial arts, basketball, swimming, golf, badminton, and table tennis. There were 398 males and 201 females. Ages ranged from 16 to 27 (mean age = 20.29, SD = 1.7).

### Instrumentation

#### Demographic questionnaire

A demographic questionnaire was used to collect the following specific information about the participants: (1) gender, (2) age, (3) sport, and (4) whether they incurred an injury in the past three weeks that could hinder their athletic performance. All demographical variables were dummy coded.

##### Connor-Davidson Resilience Scale (CD-RISC 25)

The Connor-Davidson Resilience Scale 25 (Campbell-Sills and Stein, 2007) features 25 statements to which participants respond. It measures the extent to which individuals are able to recover from, or adapt positively to personal problems, illness, pressure, failure, and painful feelings (Campbell-Sills and Stein, 2007). Each response to a statement is rated on a five-point scale from 0 (not true at all) to 4 (true nearly all the time). Higher scores indicate greater levels of perceived resilience. A sample statement from the CD-RISC 25 is, “I tend to bounce back after illness, injury, or other hardships” (Campbell-Sills and Stein, 2007; Coates et al., 2013; Gonzalez et al., 2016). The CD-RISC 25 demonstrated reasonable test-retest reliability with Chronbach’s alpha correlation coefficient.87 (Connor and Davidson, 2003). Another resilience and CD-RISC 25 study in Japanese students by Ito et al. (2009) found good internal consistency (Cronbach’s α = 0.94 and 0.90 for two samples), and good test-retest reliability of 0.94 and 0.83. The CD-RISC 25 has been validated and is a commonly used assessment of resilience in psychological research (Campbell-Sills and Stein, 2007; Zurita-Ortega et al., 2018). The original version of the CD-RISC 25 was developed specifically to assist with clinical treatment of anxiety, depression, and other mental stresses related conditions (Connor and Davidson, 2003).

The CD-RISC 25 was used in the present study, in part, due to the internal validity of the instrument specific to sport-related areas when examining resilience in athletes (Kendell and Jablensky, 2003; Campbell-Sills and Stein, 2007; Carter and Weissbrod, 2011; Gonzalez et al., 2016; Olmo et al., 2017). Sport-related studies have shown the CD-RISC 25 to be internally reliable, and to have strong element structure, and gender invariance when examining characteristics of resilience in athletes (Campbell-Sills and Stein, 2007; Gonzalez et al., 2016). The reliability coefficient of the Chinese version of CD-RISC 25 was 0.91 (Yu and Zhang, 2007). Another factor analysis study also indicates that the Chinese version of CD-RISC 25 it is the same instrument that has been modified and translated to Chinese has good reliability and validity, with internal consistency values of the three factors at 0.88, 0.80, and 0.60 (Xie et al., 2016).

##### Beck depression inventory-II (BDI-II)

Beck and colleagues developed the Beck Depression Inventory-II (BDI-II) based on the theoretical conception that negative cognitive deformity was a major cause of depression (Jackson-Koku, 2016). The BDI-II consists of a 21-item self-reported instrument that assesses depression levels in general and psychiatric populations. The BDI-II was selected for this study because of its relatively short administration time and strong psychometric support (Beck and Steer, 1996).

Reliability of the BDI-II has been established through internal consistency and test-retest analyzes. Internal consistency analysis of the scores from a sample of 500 outpatients yielded a Chronbach’s alpha correlation coefficient of 0.92 (Beck and Steer, 1996). Analysis of the scores from a sample of 120 college students yielded a Chronbach’s alpha correlation coefficient of 0.93 (Beck and Steer, 1996). These estimates indicated that the items are highly inter-related. Test-retest reliability in a sample of 26 outpatients over a one-week interval had a correlation of 0.93 (Beck and Steer, 1996), suggesting the scores are stable across seven days. Construct validity was documented through factor analysis of the scores from a sample of 500 outpatients. The analysis yielded the two factors of somatic and cognitive symptoms (Beck and Steer, 1996).

The BDI-II has been modified several times throughout the years. The 21 items were established from observation of demeanors and manifestations that are frequently reported amongst clinically depressed patients and infrequently in non-depressed patients (Jackson-Koku, 2016). Each item from the assessment has a 4-point scale from 0 (no depressed symptom) to 3 (severe symptoms). The score is calculated by adding the reported scores from each questionnaire, where the maximum score is 63 and the minimum score is 0. Higher scores indicated more severe symptoms. In non-psychiatric populations, scores over 20 suggested an individual was suffering from depression (Kendall et al., 1987). A score under 13 is termed minimal depression; 14–19 = mild depression; 20–28 = moderate depression; and 29–63 = severe depression (Beck and Steer, 1996; Jackson-Koku, 2016). The BDI-II is a widely accepted instrument for assessing the severity of depression in diagnosed patients, as well as for detecting possible depressive symptoms in the general population (Beck, 1984; Zhu et al., 2018). The Chinese version of BDI-II has demonstrated good psychometric properties of validity and reliability, the varied of reliability are Cronbach’s alpha from 0.88 to 0.92 (Wang et al., 2020).

##### Beck anxiety inventory (BAI)

The level of anxiety symptoms among participants was measured by the Beck Anxiety Inventory (BAI) (Beck and Steer, 1993). The BAI is a self-report instrument containing 21 items rated on a scale from 0 to 3 for each item. The minimum total score is 0 and the maximum score is 63. Higher scores reflect a greater likelihood that an individual is experiencing anxiety symptoms. Recommended cutoff scores were created to identify varying degrees of anxiety, with a score of 1–7 = minimal anxiety, 8–15 = mild anxiety, 16–25 = moderate anxiety, and 26–63 = severe anxiety (Beck and Steer, 1993). The BAI was selected for this study because of its relatively short administration time and strong psychometric support (Beck and Steer, 1993).

Reliability of the BAI was established through internal consistency and test-retest analyzes. Internal consistency analysis of scores on the BAI in adult samples yielded Chronbach’s alpha correlation coefficients ranging from 0.92 to 0.94, indicating high interrelatedness of the items (Beck and Steer, 1993). Test-retest reliability during a one-week interval was 0.75, indicating stability of scores over a seven-day period (Beck and Steer, 1993). Criterion validity was established through correlations with two scales designed to measure anxiety. The BAI was significantly correlated with the Hamilton Anxiety Rating Scale (*r* = 0.51; Hamilton, 1959), the mean anxiety ratings over a seven-day period in the Weekly Record of Anxiety and Depression (*r* = 0.54; Barlow and Cerny, 1988), and both the Trait (*r* = 0.47) and State (*r* = 0.58) subscales of the State-Trait Anxiety Inventory (Spielberger et al., 1970; Beck and Steer, 1993). The BAI was translated into Chinese for use in a previous study, and exhibited good psychometric properties of validity and reliability, the internal consistency is Cronbach’s α = 0.95 (Che et al., 2006).

#### Data analysis

Data analysis was conducted using IBM SPSS Statistics, version 26.0 (IBM Corp., Armonk, NY, United States). The measured model analyzes were conducted using Mplus, version 8.4 (Los Angeles, CA, United States), applying maximum likelihood estimation methods. The structural equation model was used to examine the significant mediated effect and relationship between a two-factor model in anxiety and depression, and three factor-model in resilience. SEM is a statistical technique, commonly applied in the behavioral sciences, that combines confirmatory factor analysis models, regression models, and complex path models using observed and latent variables (Hox and Bechger, 1998; Martens and Haase, 2006). In SEM, path coefficients illustrate the relationships between latent variables and/or observed variables in a path diagram. Fit statistics included the root mean square error of approximation (RMSEA) as the primary fit criterion, with an RMSEA lower than 0.06 to indicate preferable fit. Comparative Fit index (CFI), with a cut off of 0.95 or above was used to indicate a good model fit. Tucker-Lewis Index (TLI), with a cut off of 0.95 or above was used to indicate a good model fit. Standardized Root Mean Square Residual (SRMR), with a cut off of 0.08 or less was used to indicate a good model fit (Hooper et al., 2008).

**FIGURE 1:**
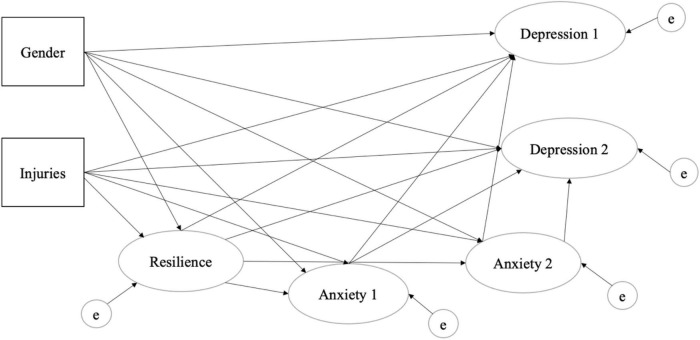
Proposed structural model between resilience, anxiety, and depression. Note: Injuries include recently reported injured male and female athletes.

Therefore, we used this method to examine whether the data from this study supported the theoretical model. Research has established that individuals with high levels of resilience are able to cope more effectively with depression and anxiety (Everly et al., 2015). Moreover, research suggested a different relationship and mediation effect exist between these constructs (Everly et al., 2015). For instance, high levels of anxiety may induce severe depression, and depression may in turn yield a lower level of resilience. On the other hand, individuals with a strong sense of resilience may be able to mitigate the symptoms of depression and anxiety. Several possible theoretical models could be proposed and compared to accommodate these constructs’ non-recursive and mediated relationship, but the hypothesized model for the study is described in Figure 1.

## Results

Descriptive statistics are shown in Table 1 for resilience, anxiety, and depression scores among all participants. Table 2 shows the correlations for categorical variables, including gender and whether or not the participants were injured.

**TABLE 1:** Descriptive statistics for resilience, anxiety and depression.

	Mean	S.D.	N
CD-RISC 25	73.59	30.746	599
BAI	24.80	5.667	599
BDI-II	23.77	6.857	599

N, sample size; S.D., standard deviation.

**TABLE 2:** Pearson correlation matrix.

		Gender	Injuries	CD-RISC 25	BAI	BDI
Gender	Pearson correlation	1				
	Sig. (2-tailed)					
	N					
Injuries	Pearson correlation	0.046	1			
	Sig. (2-tailed)	0.260				
	N					
CD-RISC 25	Pearson correlation	0.015	0.058	1		
	Sig. (2-tailed)	0.711	0.155			
	N					
BAI	Pearson correlation	–0.066	0.181**	–0.36**	1	
	Sig. (2-tailed)	0.105	<0.001	<0.001		
	N					
BDI-II	Pearson correlation	0.013	0.117*	–0.14**	0.587**	1
	Sig. (2-tailed)	0.750	0.004	<0.001	<0.001	
	N					

Sig.: Significant.

N: Sample Participants 599.

*Correlation is significant at *p* < 0.01 (two-tailed).

**Correlation is significant at *p* < 0.001 (two-tailed).

### Measurement model

#### Confirmatory factor analysis for anxiety, resilience, and depression

The initial measurement model specified the 3 latent variables of anxiety, resilience, and depression. First, each indicator was set to load only its specific latent variable and entered no indicator correlations into the initial model. Each latent variable was set so the first indicator factor loading was set at 1.0 and permitted the remainders to vary. The initial measurement model did not fit the sample data well.

The chi-square was significant, RMSEA = 0.075, which assesses the amount of model misfit (Steiger, 1990). This suggested that there was still error left unexplained by the model because the RMSEA statistic must be lower than 0.06 to indicate a good model fit. The SRMR, which is the average discrepancy between the hypothesized and observed variances and covariances in the model was 0.089. The comparative fit index (CFI) was 0.842, and the Non-normed Fit Index (NNFI) or the Tucker-Lewis index (TLI) was 0.834.

Because the initial Confirmatory Factor Analysis (CFA) measurement did not have a satisfactory model fit, necessary modifications were made in an attempt to improve the model fit indices. Modification indices from Mplus suggested the addition of error covariances between items that measured the same latent variables. Variables that exhibited the greatest change magnitude were examined first and retained in order to improve indices of model fit. Each modification added a new error covariance to those entered in the previous model. After the modification process, the final CFA on resilience, anxiety, and depression indicate much better measurement model fit results compared with the initial fit indices (See Table 3). The RMSEA in the final model was 0.035, RMESA values of.06 or less delineated a good fitting model (Steiger, 1990). The SRMR was 0.038, SRMR values of.08 or less indicated a good model fit (Hu and Bentler, 1999). The comparative fit index (CFI), compares the hypothesized model against an independence model and is ranged between 0 and 1; values above 0.95 indicate a model that fits the data well (Bentler, 1990). The CFI of 0.971 from this study was satisfactory for the model fitting results. The TLI or the NNFI analyzes the discrepancy between the chi-square value of the null model and the chi-square value of the hypothesized model, with a cutoff of 0.95 or greater demonstrating a good model fit (Tucker and Lewis, 1973). The TLI of 0.964 from this study was acceptable for the measurement model fit result.

**TABLE 3:** Fit indices for resilience, anxiety, and depression measurement model.

Model	Description	χ^2^	df	SRMR	RMSEA	CFI	TLI	*X*^2^ *diff*
Initial model	Initial measurement model	5734.210*	1422	0.046	0.071	0.868	0.852	
Final model	Final measurement model	2823.225*	1238	0.075	0.046	0.948	0.937	2910.985*

* *p* ≤ 0.001, *X*^2^ *diff*: Chi-square difference.

### Structural equation model (SEM) for resilience, anxiety, and depression

**FIGURE 2:**
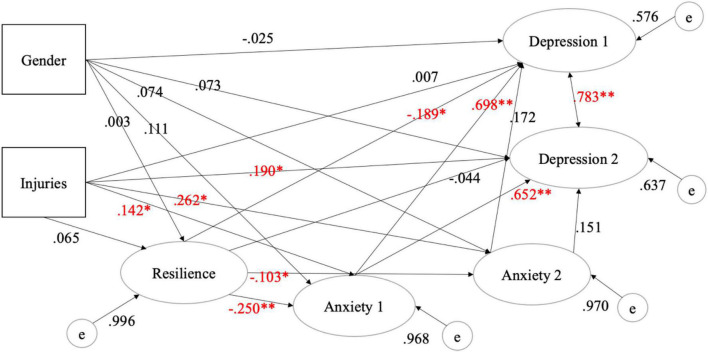
The final structural equation model on resilience, anxiety, and depression (*p ≤ 0.01, **p ≤ 0.001). Anxiety 1: cognitive factor, Anxiety 2: somatic factor, Depression 1: somatic factor, and Depression 2: cognitive factor.

A structural model was created based on the measurement model to estimate the relationships between the measured constructs of resilience, anxiety, and depression. The model fit was investigated to ensure the compatibility between the empirical evidence obtained and the model proposed. The final structural model fit indices indicated a good fit for the theoretical constructs between resilience, anxiety, and depression and are shown in Figure 2. The resultant chi-square value’s relation to the *p*-value was significant (χ2 = 2285.653; df = 1198; *p* < 0.01), CFI 0.964, TLI 0.956, RMSEA 0.039, and SRMR 0.060. All the necessary model fit indices indicated an acceptable fit for the theoretical relationship between resilience, anxiety, and depression in Chinese collegiate athletes. Based on the analysis done on the resilience, anxiety, and depression theoretical model, direct and indirect relationship between these constructs, along with categorical variables in gender effect as well as among injured and non-injured collegiate athletes were examined. The results presented in Table 4 show the estimate, standard error (SE), and two-tailed *p*-value.

**TABLE 4:** Estimate, standard error, and two-tailed *p*-value for analyzed variables.

Relationship between variables	Estimate	S.E.	Two tailed *p*-value
Anxiety 1 on resilience	–0.250	0.046	0.000**
Anxiety 2 on resilience	–0.103	0.050	0.024*
Depression 1 on resilience	–0.189	0.039	0.008*
Depression 2 on resilience	–0.044	0.037	0.238
Depression 1 on anxiety 1	0.698	0.031	0.000**
Depression 1 on anxiety 2	0.172	0.050	0.687
Depression 2 on anxiety 1	0.652	0.034	0.000**
Depression 2 on anxiety 2	0.151	0.053	0.128
Resilience on gender	0.003	0.041	0.950
Resilience on injuries	0.065	0.041	0.116
Anxiety 1 on gender	0.111	0.043	0.341
Anxiety 1 on injuries	0.142	0.045	0.002*
Anxiety 2 on gender	0.074	0.050	0.145
Anxiety 2 on injuries	0.262	0.088	0.003*
Depression 1 on gender	–0.025	0.035	0.475
Depression 1 on injuries	0.007	0.036	0.854
Depression 2 on gender	0.073	0.063	0.248
Depression 2 on injuries	0.190	0.072	0.009*

* *p* ≤ 0.01, ** *p* ≤ 0.001 *X*^2^ *diff*: Chi-square difference.

The results shown in Table 4 indicated a significant inverse relationship between anxiety and resilience and suggested that a one unit increase in resilience should correspond with a 0.103 decrease in the somatic factor and a 0.250 decrease in the cognitive factor of anxiety. The results also demonstrated a significant inverse relationship between resilience and depression. Specifically, the model predicted that athletes would experience a 0.189 decrease in the somatic factor of depression for every unit increase in resiliency level. Female and male athletes in the non-injured group did not demonstrate a statistically significant relationship between anxiety and depression, but both male and female injured athletes exhibited a statistically significant correlation between injuries, anxiety, and depression. Specifically, the model predicted that injured athletes would experience a 0.142 unit increase in the cognitive factor and a 0.262 unit increase in the somatic factor of anxiety and a 0.190 in the cognitive factor of depression. That is, an athlete who experienced an injury was more susceptible than their non-injured peers to symptoms of anxiety and depression. Anxiety and depression were positively correlated, with the model predicting that a one unit increase in the cognitive factor of anxiety would cause the athlete to experience a 0.698 and a 0.652 increase in both somatic and cognitive factors of depression. This study indicated that gender did not have a significant impact on anxiety, resilience, and depression even though a previous study pointed out that women in general tended to report higher scores in anxiety compared with men (Beck and Steer, 1993).

Results from the structural equation models provide preliminary evidence in support of the relationship between resilience, anxiety, and depression. The SEM results revealed a strong positive association between anxiety and depression among Chinese collegiate athletes. Furthermore, the SEM model found that resilience level of male and female athletes significantly predicted the degree of anxiety and depression. Additionally, the SEM results indicated that injuries had a significant impact on collegiate athletes’ levels of anxiety and depression.

## Discussion

The purpose of this study was to examine a theoretical model of resilience, anxiety, and depression in current collegiate athletes in China. The SEM model in this study was further supported: the RMSEA value was lower than 0.06; the SRMR value was lower than 0.080; and the CFI and TLI values were higher than 0.95. The SEM results thus supports the previous literature suggesting that anxiety and depression negatively affected collegiate athletes’ mental well-being. The SEM model also supported the theory regarding resilience in athletes, suggesting that athletes with higher levels of resilience had lower levels of anxiety and depression.

According to the previous literature on resilience theory, athletes’ resilience can significantly improve through the enhancement of personal skills/assets such as coping, problem solving, self-efficacy, emotional control, and intelligence. Furthermore, resilience can improve through positive perceptions of family and community support resources (Pivnick and Villegas, 2000; Wang et al., 2015; Coulombe et al., 2020). Personal skill/asset development and supportive resources are two critical factors in resilience theory that have been shown either to alleviate or minimize the negative influences of hardship and risk (Zhang, 2010). Based on the results of this and other studies, resilience education should be encouraged and used to teach athletes how to improve personal skills and develop support resources. For enhancing personal skills, a resilience education program could emphasize the ideas of positive psychology by helping athletes focus more on human virtues, potential, and motives (Zhang, 2010; Tunariu et al., 2017).

Researchers of positive psychology asserts that there are two primary factors for mental well-being. It is not just the absence of mental disorders in an individual that indicate a person has healthy psychological and emotional well-being (Suldo and Shaffer, 2008). Subjective well-being (SWB) is another factor related to mental well-being (Diener, 2000; Magalhães and Calheiros, 2017). Subjective well-being is the technical term for happiness that consists of three correlated but different constructs: life satisfaction, positive emotional affect, and negative emotional affect (Diener, 2000; Magalhães and Calheiros, 2017). Life satisfaction involves an overall and area-specific (e.g., school, job, family, social relations) cognitive assessment of an individual’s happiness. Subjective well-being promotes a positive appraisal of the overall quality of one’s life, and internally generates a continuum of positive affect (e.g., elation, delight) to overcome negative affect (anger, guilt, sorrow) (Diener, 2000; Suldo and Shaffer, 2008; Magalhães and Calheiros, 2017).

The concept of positive psychology promotes achieving higher levels of SWB or happiness in order to reinforce positive experiences that help us endure and thrive during challenging times (resilient and positive trait) (Seligman and Csikszentmihalyi, 2000). In seeking higher levels of happiness, it is useful to differentiate between two types of positive experiences: pleasurable experiences and enjoyable experiences. Pleasurable experiences refer to the feelings that come from rewarding the homeostatic needs such as appetite, thirst, sex, and other bodily comforts, and the material possessions that satisfy greed and prodigality (e.g., money, fame, houses, sport cars) (Seligman and Csikszentmihalyi, 2000; Muñiz-Velaìzquez et al., 2017). Enjoyable experiences, on the other hand, are characterized by feelings that transcend the limits of homeostasis and material desires. Enjoyable experiences in positive psychology are often described as the feeling of achieving a goal, breaking a record, performing a good deed, or performing well in sports or the arts. Enjoyment, instead of pleasure, is the foremost factor that leads to long-lasting happiness and personal growth (Seligman and Csikszentmihalyi, 2000; Muñiz-Velaìzquez et al., 2017). When we look beyond the pleasurable experiences and materialism and pursue enjoyable experiences to promote happiness, we may see things differently even in trying times. We can more easily generate the intrinsic motivation and joy to work through obstacles.

The principle of positive psychology is not new and dates back at least to the time of ancient Greece (Snyder et al., 2015). Aristotle described the Greek word *eudemonism* as the conceptual scaffold that connects the practice of virtue with human happiness (Aristotle, 2001; Snyder et al., 2015; Muñiz-Velaìzquez et al., 2017). In *The Myth of Sisyphus* author Albert Camus describes how Sisyphus’s punishment for his sins was that he was forced to roll an extremely heavy rock up a mountain, only to then watched the rock roll back to the bottom of the hill. Sisyphus is forced to repeat this action indefinitely. Though his efforts in rolling the rock to the top over and over again could be described as hard, tedious, futile, and absurd, the author attempts to convince readers that Sisyphus was in fact happy. Camus even stated: “The struggle itself toward the heights is enough to fill a man’s heart. One must imagine Sisyphus happy!” (Camus and O’Brien, 1991). The moral of this story is that it is the process of getting something done, even if that thing is hard, tragic, or even meaningless, can be enough to make us happy if we develop a positive mindset. Sisyphus may be an imaginative example of a resilient character, but in the real-world setting, there have been numerous individuals who have achieved incredible feats despite their hardships. For instance, Ludwig van Beethoven, who is revered as one of the greatest classical composers in history, composed some of his best musical works: Moonlight Sonata and Symphony Number 9 “Ode to Joy” despite becoming deaf, homeless, broke, and suicidal (Seligman and Csikszentmihalyi, 2000; Wallace, 2018). How could someone who went through such difficult times composed such cheerful and timeless pieces like “Ode to Joy”? Wallace’s book, *Hearing Beethoven: A story of musical loss and discovery*, suggests that Beethoven believed that he could still use his talent to bring good music to others and it is that belief that kept him going (Seligman and Csikszentmihalyi, 2000; Wallace, 2018). The motivation to benefit society with such masterful melodies (altruism), the love, creativity, and dedication of Beethoven, is an example of the concept of positive psychology. By pursuing positive human virtues, people may gain resilient traits that will help them overcome adversities.

Perhaps teaching college students and collegiate athletes about the concepts of positive psychology could be used to direct their thinking toward positive emotion in order to nurture their personal skills in resilience. Recommendations coming from resilience literature specific to the Chinese culture include combining traditional elements of Chinese culture with resilience education for Chinese athletes. Many characteristics of resilience are relevant to Chinese traditional culture and include elements of Taoism, Confucianism, and Collectivist culture (Xi et al., 2015). Emphasis on the values of Chinese tradition may help Chinese athletes to conceptualize the theory of resilience more effectively. By bridging the gap between traditional Chinese beliefs and the role of being a Chinese athlete the individual may be better prepared to utilize concepts of resilience when they face difficult times and undo pressure. For instance, resilience from the perspective of Confucianism is about working hard during difficult times, and finding sweetness amidst bitterness (Xi et al., 2015). Hence, sport psychologists or other practitioners could integrate aspects of traditional Chinese culture and philosophy in developing Chinese athletes’ resilience.

At the community level the resource component in resilience theory brings together coaches, teachers, parents, teammates, and others to emphasize the civic virtue that each person can be a better citizen and develop a strong work ethic, empathy, altruism, responsibility, and tolerance (Seligman and Csikszentmihalyi, 2000; Snyder et al., 2015). For the collegiate Chinese athlete specifically, coaches should put significant effort into building a positive coach-athlete relationship. Prior research has demonstrated that coaches’ support and care play significant roles in Chinese athletes’ mental health and psychological wellbeing (Choi et al., 2013; Hampson and Jowett, 2014; Yang et al., 2020). Most Chinese athletes are involved in sports through a nation-wide system, and often these athletes have lived with their coaches in a fairly closed accommodation provided by the nation-wide sport centers in each province (Liu et al., 2016). It would therefore make sense that an effective resilience education program would also provide training for coaches that emphasized the coach-athlete relationship. If more coaches in China received positive psychology education, it could be beneficial for athletes in their resilience development.

### Limitations

It is essential to highlight some of the limitations of the present study that could affect how the findings are interpreted. First, a limitation in the data may be that it was not specified whether athletes participated in team sports or individual sports, hence the analyzes did not discern whether a certain type of sport had an effect on an athlete’s level of depression, anxiety, or resilience. In addition, the study did not control for whether participants were suffering from any personal difficulties such as family struggles, romantic relationship difficulties, or recent poor sport performance outcomes, all of which could affect resilience, depression, and anxiety scores.

Additionally, data for this research were collected during the Novel Coronavirus 19 (COVID-19) pandemic, so it is also possible that this time period contributed to student athletes’ negative emotions and feelings of anxiety and depression during uncertain times. A future study should be conducted during a time when the COVID-19 pandemic is not a factor in order to ascertain what impact, if any, COVID-19 had on the findings of this study.

Additionally, this study employed a quantitative data analysis method to describe participants’ experiences related to anxiety, depression, and resilience. The quantitative approach of this study involved asking specific and narrow questions of participants and then statistically analyzing those responses to provide numerical and objective descriptions of their responses. However, solely relying on a quantitative data analysis method may not satisfactorily explain the complex relationship between psychological and subjective experiences tied to anxiety, depression, and resilience (Clark and Creswell, 2009). Accordingly, a potential shortcoming in using only Likert-type scale responses in quantitative data analysis was that nuanced information and personal experiences will likely not be reflected in these findings (Sandelowski, 2000). In other words, restricting responses to the statements presented to participants may not sufficiently represent the breadth and depth of a participant’s experience (Clark and Creswell, 2009). One of the strengths of a qualitative approach is its ability to provide participants the opportunity to express their perceptions and thoughts in their own words. Open-ended questions could engender answers that are meaningful to the participant, and thereby enable a greater understanding of the explored phenomena. Future research on similar topics should consider qualitative research methods as well as quantitative methods to gather a more holistic set of responses from participants (Merriam, 2009).

## Conclusion

Results of the present study showed a significant relationship between resilience, anxiety, and depression. Perhaps the most meaningful finding was that across both male and female athletes, individuals with greater levels of resiliency experienced lower levels of anxiety and depression. The effect of resiliency on depression and anxiety may be linked to success in future athletic, educational, or occupational pursuits (Fletcher and Sarkar, 2012).

The results of this study support other studies’ findings and can be used to encourage collegiate athletes to better cope with depression and anxiety by developing greater resiliency. The field of positive psychology asserts that positive thinking may help people tremendously to improve their resilience and overall well-being (Snyder et al., 2015). Previous literature suggests that when individuals have optimistic thoughts for extended time periods, and align their actions with those thoughts, they are more likely to achieve personal goals and become healthier and happier (Dispenza, 2012; Tunariu et al., 2017). Accordingly, universities, team leadership (coaches and administrators), and educational professionals should promote resilience theory-based education to contribute to the positive development of Chinese athletes’ mental health.

## Data availability statement

The original contributions presented in this study are included in the article/Supplementary material, further inquiries can be directed to the corresponding author.

## Ethics statement

The studies involving human participants were reviewed and approved by Lyu Chengjie, School of Physical Education, Guangzhou Sport Univeristy, China. The patients/participants provided their written informed consent to participate in this study.

## Author contributions

CL and RM first initiated the topic of the study. CL conducted all the data collection process in Guangzhou, China, and assisted RM to run the data analysis. RH and DP helped RH with for the study and helped RH to edit the writing portion for this study. All authors contributed to the article and approved the submitted version.
