# Supplementary material for: The relationship between resilience, anxiety, and depression in Chinese collegiate athletes
Source: Front Psychol. 2022 Aug 12;13:921419. doi: 10.3389/fpsyg.2022.921419 (PMC9416885; doi:10.3389/fpsyg.2022.921419)
Supplement: Supplementary file 3 [file Table_3.doc]

**广东省高校体育类学生心理健康状况调查表**

**Psychological Status/Health Questionnaires for Collegiate Athletes GuangDong Province in Mainland China**

一、基本情况 Demographic Questionnaire

1、您的性别 Gender: [单选题]

| 选项 Options | 小计 Subtotal | 比例 Ratio |
| --- | --- | --- |
| 男 Male | 398 | 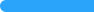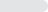66.44% |
| 女 Female | 201 | 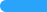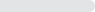33.56% |
| 本题有效填写人次  Effect Sample Size | 599 |  |

2、年龄 Age: [填空题]

填空题数据请通过下载详细数据获取

3、运动项目 Sports: [填空题]

填空题数据请通过下载详细数据获取

4、运动等级: Athletes’ rating in Sport Achievement [单选题]

| 选项 Options | 小计 Subtotal | 比例 Ratio |
| --- | --- | --- |
| 国际健将 Olympian who had achieved Medals | 4 | 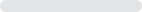0.67% |
| 国家健将 National Outstanding Athletes | 9 | 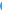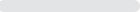1.5% |
| 国家一级 1^st^ grade Elite National Athlete | 72 | 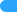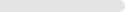12.02% |
| 国家二级 2^nd^ grade Elite National Athlete | 158 | 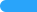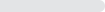26.38% |
| 国家三级 3^rd^ grade Elite National Athlete | 18 | 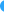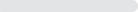3.01% |
| 无级别 High Performance Athlete | 338 | 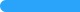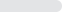56.43% |
| 本题有效填写人次 Effect Sample Size | 599 |  |

5、最近身体状况，请问最近是否承受比较严重或长期的伤病?

Your recent physical condition, do you suffer any injury or sickness in the past three weeks that could hinder your athletic performance? [填空题]

二、Beck Anxiety Inventory(贝克焦虑量表)

1 、麻痹感觉或刺痛感觉（身体上） Numbness or tingling [单选题]

| 选项 Option | 小计  Subtotal | 比例 Ratio |
| --- | --- | --- |
| 没有 Not at all | 428 | 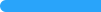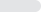71.45% |
| 轻度，但不影响 Mildly | 135 | 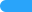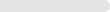22.54% |
| 中等，有时候不舒服 Moderately | 31 | 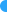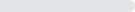5.18% |
| 重度，带给我很多困扰 Severely | 5 | 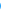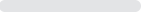0.83% |
| 本题有效填写人次 Effect Size | 599 |  |

2 、发热（感到发热） Feeling hot [单选题]

| 选项 Option | 小计  Subtotal | 比例 Ratio |
| --- | --- | --- |
| 没有 Not at all | 574 | 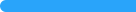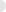95.83% |
| 轻度，但不影响 Mildly | 23 | 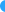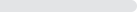3.84% |
| 中等，有时候不舒服 Moderately | 2 | 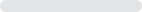0.33% |
| 重度，带给我很多困扰 Severely | 0 | 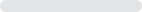0% |
| 本题有效填写人次 Effect Size | 599 |  |

3、脚颤（脚部颤抖）Wobbliness in legs [单选题]

| 选项 Option | 小计  Subtotal | 比例 Ratio |
| --- | --- | --- |
| 没有 Not at all | 510 | 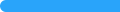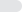85.14% |
| 轻度，但不影响 Mildly | 81 | 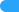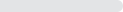13.52% |
| 中等，有时候不舒服 Moderately | 8 | 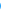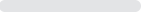1.34% |
| 重度，带给我很多困扰 Severely | 0 | 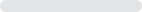0% |
| 本题有效填写人次 Effect Size | 599 |  |

4 、不能松弛（不能放松） Unable to relax [单选题]

| 选项 Option | 小计  Subtotal | 比例 Ratio |
| --- | --- | --- |
| 没有 Not at all | 478 | 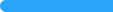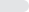79.8% |
| 轻度，但不影响 Mildly | 92 | 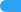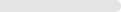15.36% |
| 中等，有时候不舒服 Moderately | 27 | 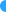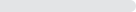4.51% |
| 重度，带给我很多困扰 Severely | 2 | 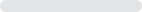0.33% |
| 本题有效填写人次 Effect Size | 599 |  |

5 、担心有最坏的事情发生 Fear of the worst happening [单选题]

| 选项 Option | 小计  Subtotal | 比例 Ratio |
| --- | --- | --- |
| 没有 Not at all | 430 | 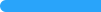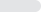71.79% |
| 轻度，但不影响 Mildly | 129 | 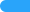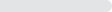21.54% |
| 中等，有时候不舒服 Moderately | 32 | 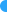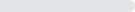5.34% |
| 重度，带给我很多困扰 Severely | 8 | 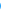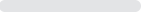1.34% |
| 本题有效填写人次 Effect Size | 599 |  |

6 、头晕、目眩或眼花 Dizzy or lightheaded [单选题]

| 选项 Option | 小计  Subtotal | 比例 Ratio |
| --- | --- | --- |
| 没有 Not at all | 486 | 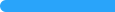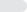81.14% |
| 轻度，但不影响 Mildly | 99 | 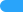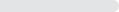16.53% |
| 中等，有时候不舒服 Moderately | 14 | 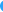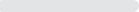2.34% |
| 重度，带给我很多困扰 Severely | 0 | 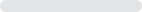0% |
| 本题有效填写人次 Effect Size | 599 |  |

7 、心动过速、心悸（心跳加快） Heart pounding or racing [单选题]

| 选项 Option | 小计  Subtotal | 比例 Ratio |
| --- | --- | --- |
| 没有 Not at all | 499 | 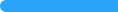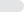83.31% |
| 轻度，但不影响 Mildly | 88 | 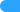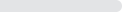14.69% |
| 中等，有时候不舒服 Moderately | 12 | 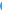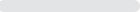2% |
| 重度，带给我很多困扰 Severely | 0 | 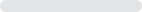0% |
| 本题有效填写人次 Effect Size | 599 |  |

8 、不安定（心神不定）Unsteady [单选题]

| 选项 Option | 小计  Subtotal | 比例 Ratio |
| --- | --- | --- |
| 没有 Not at all | 468 | 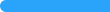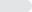78.13% |
| 轻度，但不影响 Mildly | 117 | 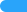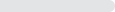19.53% |
| 中等，有时候不舒服 Moderately | 12 | 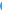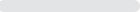2% |
| 重度，带给我很多困扰 Severely | 2 | 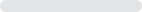0.33% |
| 本题有效填写人次 Effect Size | 599 |  |

9、 恐惧、害怕（感到惊吓） Terrified [单选题]

| 选项 Option | 小计  Subtotal | 比例 Ratio |
| --- | --- | --- |
| 没有 Not at all | 521 | 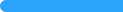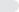86.98% |
| 轻度，但不影响 Mildly | 68 | 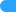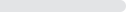11.35% |
| 中等，有时候不舒服 Moderately | 9 | 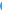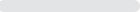1.5% |
| 重度，带给我很多困扰 Severely | 1 | 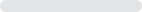0.17% |
| 本题有效填写人次 Effect Size | 599 |  |

10 、紧张 Nervous [单选题]

| 选项 Option | 小计  Subtotal | 比例 Ratio |
| --- | --- | --- |
| 没有 Not at all | 418 | 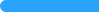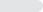69.78% |
| 轻度，但不影响 Mildly | 152 | 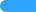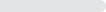25.38% |
| 中等，有时候不舒服 Moderately | 26 | 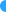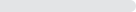4.34% |
| 重度，带给我很多困扰 Severely | 3 | 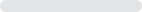0.5% |
| 本题有效填写人次 Effect Size | 599 |  |

11 、窒息感（快要不能呼吸的感觉） Feelings of choking [单选题]

| 选项 Option | 小计  Subtotal | 比例 Ratio |
| --- | --- | --- |
| 没有 Not at all | 544 | 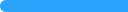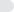90.82% |
| 轻度，但不影响 Mildly | 42 | 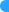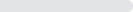7.01% |
| 中等，有时候不舒服 Moderately | 12 | 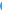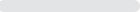2% |
| 重度，带给我很多困扰 Severely | 1 | 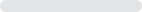0.17% |
| 本题有效填写人次 Effect Size | 599 |  |

12 、手发抖 Hands trembling [单选题]

| 选项 Option | 小计  Subtotal | 比例 Ratio |
| --- | --- | --- |
| 没有 Not at all | 520 | 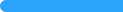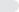86.81% |
| 轻度，但不影响 Mildly | 69 | 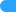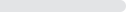11.52% |
| 中等，有时候不舒服 Moderately | 10 | 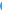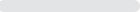1.67% |
| 重度，带给我很多困扰 Severely | 0 | 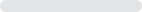0% |
| 本题有效填写人次 Effect Size | 599 |  |

13 、摇摆、震动、不安定（摇晃） Shaky [单选题]

| 选项 Option | 小计  Subtotal | 比例 Ratio |
| --- | --- | --- |
| 没有 Not at all | 562 | 93.82% |
| 轻度，但不影响 Mildly | 32 | 5.34% |
| 中等，有时候不舒服 Moderately | 5 | 0.83% |
| 重度，带给我很多困扰 Severely | 0 | 0% |
| 本题有效填写人次 Effect Size | 599 |  |

14 、害怕失去控制 Fear of losing control [单选题]

| 选项 Option | 小计  Subtotal | 比例 Ratio |
| --- | --- | --- |
| 没有 Not at all | 528 | 88.15% |
| 轻度，但不影响 Mildly | 54 | 9.02% |
| 中等，有时候不舒服 Moderately | 14 | 2.34% |
| 重度，带给我很多困扰 Severely | 3 | 0.5% |
| 本题有效填写人次 Effect Size | 599 |  |

15 、呼吸困难 Difficulty breathing [单选题]

| 选项 Option | 小计  Subtotal | 比例 Ratio |
| --- | --- | --- |
| 没有 Not at all | 547 | 91.32% |
| 轻度，但不影响 Mildly | 42 | 7.01% |
| 中等，有时候不舒服 Moderately | 9 | 1.5% |
| 重度，带给我很多困扰 Severely | 1 | 0.17% |
| 本题有效填写人次 Effect Size | 599 |  |

16 、死亡恐怖（害怕快要死去）Fear of dying [单选题]

| 选项 Option | 小计  Subtotal | 比例 Ratio |
| --- | --- | --- |
| 没有 Not at all | 560 | 93.49% |
| 轻度，但不影响 Mildly | 29 | 4.84% |
| 中等，有时候不舒服 Moderately | 9 | 1.5% |
| 重度，带给我很多困扰 Severely | 1 | 0.17% |
| 本题有效填写人次 Effect Size | 599 |  |

17 、恐惧（感到恐慌） Scared [单选题]

| 选项 Option | 小计  Subtotal | 比例 Ratio |
| --- | --- | --- |
| 没有 Not at all | 549 | 91.65% |
| 轻度，但不影响 Mildly | 42 | 7.01% |
| 中等，有时候不舒服 Moderately | 6 | 1% |
| 重度，带给我很多困扰 Severely | 2 | 0.33% |
| 本题有效填写人次 Effect Size | 599 |  |

18 、消化不良 Indigestion or discomfort in abdomen [单选题]

| 选项 Option | 小计  Subtotal | 比例 Ratio |
| --- | --- | --- |
| 没有 Not at all | 470 | 78.46% |
| 轻度，但不影响 Mildly | 106 | 17.7% |
| 中等，有时候不舒服 Moderately | 20 | 3.34% |
| 重度，带给我很多困扰 Severely | 3 | 0.5% |
| 本题有效填写人次 Effect Size | 599 |  |

19 、晕厥、头晕、眼花 Faint [单选题]

| 选项 Option | 小计  Subtotal | 比例 Ratio |
| --- | --- | --- |
| 没有 Not at all | 539 | 89.98% |
| 轻度，但不影响 Mildly | 50 | 8.35% |
| 中等，有时候不舒服 Moderately | 10 | 1.67% |
| 重度，带给我很多困扰 Severely | 0 | 0% |
| 本题有效填写人次 Effect Size | 599 |  |

20 、脸发热、潮红 Face flushed [单选题]

| 选项 Option | 小计  Subtotal | 比例 Ratio |
| --- | --- | --- |
| 没有 Not at all | 549 | 91.65% |
| 轻度，但不影响 Mildly | 43 | 7.18% |
| 中等，有时候不舒服 Moderately | 6 | 1% |
| 重度，带给我很多困扰 Severely | 1 | 0.17% |
| 本题有效填写人次 Effect Size | 599 |  |

21 、出汗（但不是因为天气热冒汗）Sweating (not due to heat) [单选题]

| 选项 Option | 小计  Subtotal | 比例 Ratio |
| --- | --- | --- |
| 没有 Not at all | 505 | 84.31% |
| 轻度，但不影响 Mildly | 74 | 12.35% |
| 中等，有时候不舒服 Moderately | 17 | 2.84% |
| 重度，带给我很多困扰 Severely | 3 | 0.5% |
| 本题有效填写人次 Effect Size | 599 |  |

三、Beck Depression Inventory II （贝克忧郁量表第二版）

1 、关于“心情” Sadness [单选题]

| 选项 Option | 小计  Subtotal | 比例 Ratio |
| --- | --- | --- |
| 我不觉得悲伤 I do not feel sad | 515 | 85.98% |
| 很多时候我都感到悲伤  I feel sad much of the time | 81 | 13.52% |
| 我始终感觉悲伤，不能自己  I am sad all the time | 1 | 0.17% |
| 我太悲伤或不愉快，不堪忍受  I am so sad or unhappy that I can’t stand it | 2 | 0.33% |
| 本题有效填写人次 Effect Size | 599 |  |

2 、关于“未来” Pessimism [单选题]

| 选项 Option | 小计  Subtotal | 比例 Ratio |
| --- | --- | --- |
| 我对将来并不失望  I am not discouraged about my future | 553 | 92.32% |
| 对未来我感到心灰意冷  I feel more discouraged about my future that I used to be | 35 | 5.84% |
| 我感到全景暗淡  I do not expect things to work out for me | 6 | 1% |
| 我觉得将来毫无希望，无法改善  I feel my future is hopeless and will only get worse | 5 | 0.83% |
| 本题有效填写人次 Effect Size | 599 |  |

3 、关于“成败” Past failure [单选题]

| 选项 Option | 小计  Subtotal | 比例 Ratio |
| --- | --- | --- |
| 我没有感到失败 I do not feel like a failure | 462 | 77.13% |
| 我觉得比一般人失败要多一些  I have failed more than I should have | 95 | 15.86% |
| 回首往事，我看到的是很多次失败  As I look back, I see a lot of failure | 38 | 6.34% |
| 我觉得我是一个完全失败的人  I feel I am a total failure as a person | 4 | 0.67% |
| 本题有效填写人次 Effect Size | 599 |  |

4 、关于“满足感” Loss of pleasure [单选题]

| 选项 Option | 小计  Subtotal | 比例 Ratio |
| --- | --- | --- |
| 我和以前一样，从各种事情中得到满足  I get as much pleasure as I ever did from the things, I enjoy | 464 | 77.46% |
| 我不能像从前一样从各种事情中得到满足  I don’t enjoy things as much as I used to | 105 | 17.53% |
| 我从过去喜欢的事情中获得的快乐很少  I get very little pleasure from things I used to enjoy | 28 | 4.67% |
| 我对一切事情都不满意或感到枯草无味  I can’t little pleasure from things I used to enjoy | 2 | 0.33% |
| 本题有效填写人次 Effect Size | 599 |  |

5 、关于“负罪感” Guilty feelings [单选题]

| 选项 Option | 小计  Subtotal | 比例 Ratio |
| --- | --- | --- |
| 我不感到罪过 I don’t fell particularly guilty | 499 | 83.31% |
| 我在相当部分的时间里感到罪过  I feel guilty over many things I have done or should have done | 90 | 15.03% |
| 我在大部分时间里觉得有罪  I feel quite guilty most of the time | 6 | 1% |
| 我在任何时候都觉得有罪  I feel guilty all of the time | 4 | 0.67% |
| 本题有效填写人次 Effect Size | 599 |  |

6 、关于“惩罚” Punishment feelings [单选题]

| 选项 Option | 小计  Subtotal | 比例 Ratio |
| --- | --- | --- |
| 我没有觉得受到惩罚  I don’t feel I am being punished | 478 | 79.8% |
| 我觉得可能受到惩罚  I feel I may be punished | 97 | 16.19% |
| 我觉得自己会受到惩罚  I expect to be punished | 12 | 2% |
| 我觉得正在受到惩罚  I feel I am being punished | 12 | 2% |
| 本题有效填写人次 Effect Size | 599 |  |

7 、关于“自己” Self-dislike [单选题]

| 选项 Option | 小计  Subtotal | 比例 Ratio |
| --- | --- | --- |
| 我对自己并不失望  I feel the same about myself as ever | 506 | 84.47% |
| 我对自己感到失望  I have lost confidence in myself | 78 | 13.02% |
| 我对自己感到讨厌  I am disappointed in myself | 11 | 1.84% |
| 我恨我自己  I dislike myself | 4 | 0.67% |
| 本题有效填写人次 Effect Size | 599 |  |

8 、在“自我评价”方面 Self-criticalness [单选题]

| 选项 Option | 小计  Subtotal | 比例 Ratio |
| --- | --- | --- |
| 我觉得我并不比其他人更不好  I don’t criticize or blame myself more than usual | 521 | 86.98% |
| 我比过去责备自己更多  I am more critical or myself than I used to be | 70 | 11.69% |
| 我在所有的时间里都责备自己的过错  I criticize myself for all of my faults | 4 | 0.67% |
| 我责备自己所有的事情都弄坏了  I criticize myself for everything bad that happens | 4 | 0.67% |
| 本题有效填写人次 Effect Size | 599 |  |

9 、关于“哭” Crying [单选题]

| 选项 Option | 小计  Subtotal | 比例 Ratio |
| --- | --- | --- |
| 我哭泣和往常一样  I don’t cry any more than I used to | 468 | 78.13% |
| 我比往常哭的多  I cry more than I used to | 24 | 4.01% |
| 现在任何小事情都会让我哭  I cry overevery little thing | 13 | 2.17% |
| 我过去能哭，但现在要哭也哭不出来  I feel like crying, but I can’t | 94 | 15.69% |
| 本题有效填写人次 Effect Size | 599 |  |

10、关于“生气” Agitation [单选题]

| 选项 Option | 小计  Subtotal | 比例 Ratio |
| --- | --- | --- |
| 和过去相比，我现在生气并不多  I am no more restless or would up than usual | 531 | 88.65% |
| 我现在比往常更容易生气发火  I feel more restless or wound up than usual | 56 | 9.35% |
| 我觉得现在所有的事都容易让我生气  I am so restless or agitated that it’s hard to stay still | 4 | 0.67% |
| 我非常烦躁不安，必须不停走动或做事情  I am so restless or agitated that I have to keep moving or doing something | 8 | 1.34% |
| 本题有效填写人次 Effect Size | 599 |  |

11 、失去对事物的兴趣 Loss of Interest [单选题]

| 选项 Option | 小计  Subtotal | 比例 Ratio |
| --- | --- | --- |
| 我对其他人和事没有失去兴趣  I have not lost interest in other people or activities | 478 | 79.8% |
| 我对其他人和事失去一定的兴趣  I am less interested in other people or things than before | 107 | 17.86% |
| 我已对其他人的事失去兴趣  I have lost most of my interest in other people or things. | 9 | 1.5% |
| 我发觉自己对任何事情都没有兴趣  It’s hard to get interested in anything | 5 | 0.83% |
| 本题有效填写人次 Effect Size | 599 |  |

12、关于“做决定” Indecisiveness [单选题]

| 选项 Option | 小计  Subtotal | 比例 Ratio |
| --- | --- | --- |
| 和过往相比，我的决策能力还是一样  I make decisions about as well as ever | 510 | 85.14% |
| 我最近感觉难以作出决定  I find it more difficult to make decisions than usual | 63 | 10.52% |
| 和以往相比，我很难做出决定  I have much greater difficulty in making decisions than I used to | 15 | 2.5% |
| 我有困难做出任何重要决定  I have trouble making any decisions | 11 | 1.84% |
| 本题有效填写人次 Effect Size | 599 |  |

13、在“价值”方面 Worthlessness [单选题]

| 选项 Option | 小计  Subtotal | 比例 Ratio |
| --- | --- | --- |
| 我觉得自己跟以前一样有价值  I do not feel I am worthless | 515 | 85.98% |
| 我认为自己不如过去有价值或有用了  I don’t consider myself as worthwhile and useful as I used to | 55 | 9.18% |
| 我觉得自己不如别人有价值  I feel more worthless as compared to other people | 22 | 3.67% |
| 我觉得自己毫无价值  I feel utterly worthless | 7 | 1.17% |
| 本题有效填写人次 Effect Size | 599 |  |

14、有关于 “精力”的感觉 Loss of Energy [单选题]

| 选项 Option | 小计  Subtotal | 比例 Ratio |
| --- | --- | --- |
| 我和以前一样有很多精力和活力  I have as much energy as ever | 433 | 72.29% |
| 相较之前，我活力和精力有所下降  I have less energy than I used to have | 150 | 25.04% |
| 我没有足够精力去做很多事情  I don’t have enough energy to do very much | 13 | 2.17% |
| 我没有精力去做任何事情  I don’t have enough energy to do anything | 3 | 0.5% |
| 本题有效填写人次 Effect Size | 599 |  |

15、 关于我的“睡眠”质量 Changes in Sleeping Pattern [单选题]

| 选项 Option | 小计  Subtotal | 比例 Ratio |
| --- | --- | --- |
| 我睡觉与往常一样好  I have not experienced any change in my sleeping pattern | 407 | 67.95% |
| 我睡觉不如过去好  I don’t sleep as well as before | 144 | 24.04% |
| 我的睡眠比以前少了很多，或多了很多  I sleep a lot more/less than usual | 40 | 6.68% |
| 我比往常早醒几个小时，之后便不能再睡  I wake up 1-2 hours early and can’t get back to sleep | 8 | 1.34% |
| 本题有效填写人次 Effect Size | 599 |  |

16、关于我的“精神状态” Irritability [单选题]

| 选项 Option | 小计  Subtotal | 比例 Ratio |
| --- | --- | --- |
| 我并不感到比往常更疲乏  I am no more irritable than usual | 434 | 72.45% |
| 我比过去更容易感到疲乏  I am more irritable than usual | 147 | 24.54% |
| 几乎不管做什么，我都感到疲乏无力  I am much more irritable than usual | 17 | 2.84% |
| 我太疲乏无力，不能做任何事情  I am irritable all the time | 1 | 0.17% |
| 本题有效填写人次 Effect Size | 599 |  |

17、关于我的“食欲” Change in Appetite [单选题]

| 选项 Option | 小计  Subtotal | 比例 Ratio |
| --- | --- | --- |
| 我的食欲与往常一样  I have not experienced any change in my appetite | 499 | 83.31% |
| 我的食欲不如过去好  My appetite is not as well as before | 71 | 11.85% |
| 我现在的食欲差得多了  My appetite is very bad now | 20 | 3.34% |
| 我完全没有食欲，或总是非常渴望吃东西  I have no appetite at all | 9 | 1.5% |
| 本题有效填写人次 Effect Size | 599 |  |

18、关于“集中精神” Concentration Difficulty [单选题]

| 选项 Option | 小计  Subtotal | 比例 Ratio |
| --- | --- | --- |
| 我和过去一样可以集中精神  I can concentrate as well as ever | 476 | 79.47% |
| 我无法像过去一样集中精神  I can’t concentrate as well as usual | 102 | 17.03% |
| 任何事情都很难让我长时间集中精神  It’s hard to keep my mind on anything for very long | 18 | 3.01% |
| 任何事情都无法让我集中精神  I find I can’t concentrate on anything | 3 | 0.5% |
| 本题有效填写人次 Effect Size | 599 |  |

19、关于我的“健康状况” Tiredness or Fatigue [单选题]

| 选项 Option | 小计  Subtotal | 比例 Ratio |
| --- | --- | --- |
| 我没觉得比过去累或乏力  I am no more tired or fatigued than usual | 447 | 74.62% |
| 我比过去更容易累或乏力  I get more tired or fatigued more easily than usual | 131 | 21.87% |
| 因为太累或者太乏力，许多过去常做得事情不能做了  I get more tired or fatigued to do a lot of the things I used to do | 19 | 3.17% |
| 因为太累或者太乏力，让我无法正常生活  I am too tired or fatigued to do most of the things I used to do | 2 | 0.33% |
| 本题有效填写人次 Effect Size | 599 |  |

四、Connor Davidson Resilience Scale （心理弹性量表）

1、 我能够有效适应当事情有重大的变化

I am able to adapt when changes occur. [单选题]

| 选项 Option | 小计  Subtotal | 比例 Ratio |
| --- | --- | --- |
| 完全不是真的 Not true at all | 185 | 30.88% |
| 偶尔我同意 Rarely true | 141 | 23.54% |
| 有些时候我同意 Sometimes true | 114 | 19.03% |
| 经常的时候我同意 Often true | 61 | 10.18% |
| 绝大部分的时候我同意  True nearly all the time | 98 | 16.36% |
| 本题有效填写人次 Effect Size | 599 |  |

2、 我至少有一个亲人或好朋友为我排忧解难

I have at least one close and secure relationship that helps me when I am stressed. [单选题]

| 选项 Option | 小计  Subtotal | 比例 Ratio |
| --- | --- | --- |
| 完全不是真的 Not true at all | 149 | 24.87% |
| 偶尔我同意 Rarely true | 126 | 21.04% |
| 有些时候我同意 Sometimes true | 92 | 15.36% |
| 经常的时候我同意 Often true | 74 | 12.35% |
| 绝大部分的时候我同意  True nearly all the time | 158 | 26.38% |
| 本题有效填写人次 Effect Size | 599 |  |

3、我可以克服任何困难 I can deal with whatever comes my way. [单选题]

| 选项 Option | 小计  Subtotal | 比例 Ratio |
| --- | --- | --- |
| 完全不是真的 Not true at all | 145 | 24.21% |
| 偶尔我同意 Rarely true | 128 | 21.37% |
| 有些时候我同意 Sometimes true | 117 | 19.53% |
| 经常的时候我同意 Often true | 96 | 16.03% |
| 绝大部分的时候我同意  True nearly all the time | 113 | 18.86% |
| 本题有效填写人次 Effect Size | 599 |  |

4、 过往成功的经历给予我信心去面对新的挑战和困境

Past successes give me confidence in dealing with new challenges and difficulties. [单选题]

| 选项 Option | 小计  Subtotal | 比例 Ratio |
| --- | --- | --- |
| 完全不是真的 Not true at all | 135 | 22.54% |
| 偶尔我同意 Rarely true | 116 | 19.37% |
| 有些时候我同意 Sometimes true | 97 | 16.19% |
| 经常的时候我同意 Often true | 107 | 17.86% |
| 绝大部分的时候我同意  True nearly all the time | 144 | 24.04% |
| 本题有效填写人次 Effect Size | 599 |  |

5、 我尝试发掘幽默的一面当我面对复杂的问题

I try to see the humorous side of things when I am faced with problems. [单选题]

| 选项 Option | 小计  Subtotal | 比例 Ratio |
| --- | --- | --- |
| 完全不是真的 Not true at all | 133 | 22.2% |
| 偶尔我同意 Rarely true | 131 | 21.87% |
| 有些时候我同意 Sometimes true | 121 | 20.2% |
| 经常的时候我同意 Often true | 94 | 15.69% |
| 绝大部分的时候我同意  True nearly all the time | 120 | 20.03% |
| 本题有效填写人次 Effect Size | 599 |  |

6、 应对困难和压力的经历能够帮助我成长。

Having to cope with stress can make me stronger. [单选题]

| 选项 Option | 小计  Subtotal | 比例 Ratio |
| --- | --- | --- |
| 完全不是真的 Not true at all | 128 | 21.37% |
| 偶尔我同意 Rarely true | 94 | 15.69% |
| 有些时候我同意 Sometimes true | 105 | 17.53% |
| 经常的时候我同意 Often true | 103 | 17.2% |
| 绝大部分的时候我同意  True nearly all the time | 169 | 28.21% |
| 本题有效填写人次 Effect Size | 599 |  |

7、 我的恢复能力较强无论当我受到病痛，重伤和其他挫折的困扰。

I tend to bounce back after illness, injury, or other hardships. [单选题]

| 选项 Option | 小计  Subtotal | 比例 Ratio |
| --- | --- | --- |
| 完全不是真的 Not true at all | 139 | 23.21% |
| 偶尔我同意 Rarely true | 122 | 20.37% |
| 有些时候我同意 Sometimes true | 107 | 17.86% |
| 经常的时候我同意 Often true | 90 | 15.03% |
| 绝大部分的时候我同意  True nearly all the time | 141 | 23.54% |
| 本题有效填写人次 Effect Size | 599 |  |

8、无论结果怎样，我都会尽自己最大努力。

I give my best effort no matter what the outcome may be. [单选题]

| 选项 Option | 小计  Subtotal | 比例 Ratio |
| --- | --- | --- |
| 完全不是真的 Not true at all | 123 | 20.53% |
| 偶尔我同意 Rarely true | 91 | 15.19% |
| 有些时候我同意 Sometimes true | 84 | 14.02% |
| 经常的时候我同意 Often true | 102 | 17.03% |
| 绝大部分的时候我同意  True nearly all the time | 199 | 33.22% |
| 本题有效填写人次 Effect Size | 599 |  |

9、 我总是全力以赴去做每一件事无论结局如何

I always give my best effort in each task. [单选题]

| 选项 Option | 小计  Subtotal | 比例 Ratio |
| --- | --- | --- |
| 完全不是真的 Not true at all | 119 | 19.87% |
| 偶尔我同意 Rarely true | 97 | 16.19% |
| 有些时候我同意 Sometimes true | 99 | 16.53% |
| 经常的时候我同意 Often true | 109 | 18.2% |
| 绝大部分的时候我同意  True nearly all the time | 175 | 29.22% |
| 本题有效填写人次 Effect Size | 599 |  |

10、我能实现自己的目标

I believe I can achieve my goals, even if there are obstacles. [单选题]

| 选项 Option | 小计  Subtotal | 比例 Ratio |
| --- | --- | --- |
| 完全不是真的 Not true at all | 118 | 19.7% |
| 偶尔我同意 Rarely true | 108 | 18.03% |
| 有些时候我同意 Sometimes true | 127 | 21.2% |
| 经常的时候我同意 Often true | 109 | 18.2% |
| 绝大部分的时候我同意  True nearly all the time | 137 | 22.87% |
| 本题有效填写人次 Effect Size | 599 |  |

11、 尽管许多事看似希望渺亡，我从来都不会放弃

Even when things look hopeless, I don’t give up.

[单选题]

| 选项 Option | 小计  Subtotal | 比例 Ratio |
| --- | --- | --- |
| 完全不是真的 Not true at all | 125 | 20.87% |
| 偶尔我同意 Rarely true | 105 | 17.53% |
| 有些时候我同意 Sometimes true | 101 | 16.86% |
| 经常的时候我同意 Often true | 119 | 19.87% |
| 绝大部分的时候我同意  True nearly all the time | 149 | 24.87% |
| 本题有效填写人次 Effect Size | 599 |  |

12、当自己陷入困境，我知道如何寻找帮助

During times of stress/crisis, I know where to turn for help. [单选题]

| 完全不是真的 Not true at all | 114 | 19.03% |
| --- | --- | --- |
| 偶尔我同意 Rarely true | 113 | 18.86% |
| 有些时候我同意 Sometimes true | 129 | 21.54% |
| 经常的时候我同意 Often true | 107 | 17.86% |
| 绝大部分的时候我同意  True nearly all the time | 136 | 22.7% |
| 本题有效填写人次 Effect Size | 599 |  |

13、 巨大压力下，我思维清晰并注意力集中

Under pressure, I stay focused and think clearly. [单选题]

| 选项 Option | 小计  Subtotal | 比例 Ratio |
| --- | --- | --- |
| 完全不是真的 Not true at all | 115 | 19.2% |
| 偶尔我同意 Rarely true | 118 | 19.7% |
| 有些时候我同意 Sometimes true | 133 | 22.2% |
| 经常的时候我同意 Often true | 99 | 16.53% |
| 绝大部分的时候我同意  True nearly all the time | 134 | 22.37% |
| 本题有效填写人次 Effect Size | 599 |  |

14、 我宁愿自己主动去解决问题，都不想等他人作出决定

I prefer to take the lead in solving problems rather than letting others make all the decisions. [单选题]

| 选项 Option | 小计  Subtotal | 比例 Ratio |
| --- | --- | --- |
| 完全不是真的 Not true at all | 114 | 19.03% |
| 偶尔我同意 Rarely true | 106 | 17.7% |
| 有些时候我同意 Sometimes true | 127 | 21.2% |
| 经常的时候我同意 Often true | 100 | 16.69% |
| 绝大部分的时候我同意  True nearly all the time | 152 | 25.38% |
| 本题有效填写人次 Effect Size | 599 |  |

15、 面对挫折和失败，我永不气馁

I am not easily discouraged by failure. [单选题]

| 选项 Option | 小计  Subtotal | 比例 Ratio |
| --- | --- | --- |
| 完全不是真的 Not true at all | 108 | 18.03% |
| 偶尔我同意 Rarely true | 101 | 16.86% |
| 有些时候我同意 Sometimes true | 111 | 18.53% |
| 经常的时候我同意 Often true | 119 | 19.87% |
| 绝大部分的时候我同意  True nearly all the time | 160 | 26.71% |
| 本题有效填写人次 Effect Size | 599 |  |

16、 我认为自己有出色的能力去面对人生困境和挑战

I think of myself as a strong person when dealing with life’s challenges and difficulties. [单选题]

| 选项 Option | 小计  Subtotal | 比例 Ratio |
| --- | --- | --- |
| 完全不是真的 Not true at all | 112 | 18.7% |
| 偶尔我同意 Rarely true | 111 | 18.53% |
| 有些时候我同意 Sometimes true | 119 | 19.87% |
| 经常的时候我同意 Often true | 96 | 16.03% |
| 绝大部分的时候我同意  True nearly all the time | 161 | 26.88% |
| 本题有效填写人次 Effect Size | 599 |  |

17、 必要时, 我可以作出果断决择虽然我的抉择或会影响到他人

I can make unpopular or difficult decisions that affect other people, if it is necessary. [单选题]

| 选项 Option | 小计  Subtotal | 比例 Ratio |
| --- | --- | --- |
| 完全不是真的 Not true at all | 112 | 18.7% |
| 偶尔我同意 Rarely true | 114 | 19.03% |
| 有些时候我同意 Sometimes true | 139 | 23.21% |
| 经常的时候我同意 Often true | 91 | 15.19% |
| 绝大部分的时候我同意  True nearly all the time | 143 | 23.87% |
| 本题有效填写人次 Effect Size | 599 |  |

18、 我可以承受不愉快或痛苦的经历和情感例如悲伤，恐惧，和愤怒

I am able to handle unpleasant or painful feelings like sadness, fear, and anger. [单选题]

| 选项 Option | 小计  Subtotal | 比例 Ratio |
| --- | --- | --- |
| 完全不是真的 Not true at all | 122 | 20.37% |
| 偶尔我同意 Rarely true | 98 | 16.36% |
| 有些时候我同意 Sometimes true | 126 | 21.04% |
| 经常的时候我同意 Often true | 90 | 15.03% |
| 绝大部分的时候我同意  True nearly all the time | 163 | 27.21% |
| 本题有效填写人次 Effect Size | 599 |  |

19、 有时候在应对人生困境时，我们要相信自己的直觉并付诸于行动

In dealing with life’s problems, sometimes you have to act on a hunch without knowing why. [单选题]

| 选项 Option | 小计  Subtotal | 比例 Ratio |
| --- | --- | --- |
| 完全不是真的 Not true at all | 114 | 19.03% |
| 偶尔我同意 Rarely true | 94 | 15.69% |
| 有些时候我同意 Sometimes true | 129 | 21.54% |
| 经常的时候我同意 Often true | 111 | 18.53% |
| 绝大部分的时候我同意  True nearly all the time | 151 | 25.21% |
| 本题有效填写人次 Effect Size | 599 |  |

20、 我有重要的人生意义

I have a strong sense of purpose in life. [单选题]

| 选项 Option | 小计  Subtotal | 比例 Ratio |
| --- | --- | --- |
| 完全不是真的 Not true at all | 110 | 18.36% |
| 偶尔我同意 Rarely true | 84 | 14.02% |
| 有些时候我同意 Sometimes true | 111 | 18.53% |
| 经常的时候我同意 Often true | 110 | 18.36% |
| 绝大部分的时候我同意  True nearly all the time | 184 | 30.72% |
| 本题有效填写人次 Effect Size | 599 |  |

21、 我感觉游刃有如地掌控自己的生活

I feel in control of my life. [单选题]

| 选项 Option | 小计  Subtotal | 比例 Ratio |
| --- | --- | --- |
| 完全不是真的 Not true at all | 123 | 20.53% |
| 偶尔我同意 Rarely true | 115 | 19.2% |
| 有些时候我同意 Sometimes true | 129 | 21.54% |
| 经常的时候我同意 Often true | 101 | 16.86% |
| 绝大部分的时候我同意  True nearly all the time | 131 | 21.87% |
| 本题有效填写人次 Effect Size | 599 |  |

22 、我喜欢挑战自己 I like challenges. [单选题]

| 选项 Option | 小计  Subtotal | 比例 Ratio |
| --- | --- | --- |
| 完全不是真的 Not true at all | 114 | 19.03% |
| 偶尔我同意 Rarely true | 101 | 16.86% |
| 有些时候我同意 Sometimes true | 134 | 22.37% |
| 经常的时候我同意 Often true | 100 | 16.69% |
| 绝大部分的时候我同意  True nearly all the time | 150 | 25.04% |
| 本题有效填写人次 Effect Size | 599 |  |

23、 我努力去追求自己的理想尽管我认识到中间有很多障碍

I work to attain my goals no matter what roadblocks I encounter in my achievements. [单选题]

| 选项 Option | 小计  Subtotal | 比例 Ratio |
| --- | --- | --- |
| 完全不是真的 Not true at all | 118 | 19.7% |
| 偶尔我同意 Rarely true | 93 | 15.53% |
| 有些时候我同意 Sometimes true | 126 | 21.04% |
| 经常的时候我同意 Often true | 107 | 17.86% |
| 绝大部分的时候我同意  True nearly all the time | 155 | 25.88% |
| 本题有效填写人次 Effect Size | 599 |  |

24、 对自己以往的成功经历而自豪

I take pride in my achievements. [单选题]

| 选项 Option | 小计  Subtotal | 比例 Ratio |
| --- | --- | --- |
| 完全不是真的 Not true at all | 109 | 18.2% |
| 偶尔我同意 Rarely true | 102 | 17.03% |
| 有些时候我同意 Sometimes true | 148 | 24.71% |
| 经常的时候我同意 Often true | 90 | 15.03% |
| 绝大部分的时候我同意  True nearly all the time | 150 | 25.04% |
| 本题有效填写人次 Effect Size | 599 |  |
